# Supplementary material for: Mucosal prime-boost immunization with live murine pneumonia virus-vectored SARS-CoV-2 vaccine is protective in macaques
Source: Nat Commun. 2024 Apr 26;15:3553. doi: 10.1038/s41467-024-47784-6 (PMC11053155; doi:10.1038/s41467-024-47784-6)
Supplement: Supplementary file 1 — Supplementary Information [file 41467_2024_47784_MOESM1_ESM.pdf]

Supplementary Information for

Mucosal prime-boost immunization with live murine pneumonia virus-vectored SARS-CoV-2 vaccine is protective in macaques

Jaclyn A. Kaiser, Christine E. Nelson, Xueqiao Liu, Hong-Su Park, Yumiko Matsuoka, Cindy Luongo, Celia Santos, Laura R. H. Ahlers, Richard Herbert, Ian N. Moore, Temeri Wilder-Kofie, Rashida Moore, April Walker, Lijuan Yang, Shirin Munir, I-Ting Teng, Peter D. Kwong, Kennichi Dowdell, Hanh Nguyen, JungHyun Kim, Jeffrey I. Cohen, Reed F. Johnson, Nicole L. Garza, Laura E. Via, Daniel L. Barber, Ursula J. Buchholz[1], Cyril Le Nouën[1]

[1] E-mail: ubuchholz@niaid.nih.gov; lenouenc@niaid.nih.gov

This file includes:

Supplementary Methods 1 to 2

Supplementary Figures S1 to S7

Supplementary References 1 to 5

Supplementary Methods 1. Expression and purification of S and RBD probes for identification of S-specific B cells.

The S probe was expressed from a cDNA that encodes for the prefusion stabilized single-chain Fc tagged SARS-CoV-2 S protein (S-2P, derived from the ancestral Wuhan-1 sequence), conjugated to an Avi tag [1]. This construct was transfected into Expi293 cells and on day 6 post-transfection, the supernatant was harvested, and cells and debris were removed by centrifugation, followed by filtration through a 0.2 μm PES membrane. Then, the supernatant was incubated with Protein A Fast Flow resin (Cytiva) for 2 hours at 4°C with rotation. The cell supernatant/resin mixture was poured onto gravity flow columns (BioRad, cat# 7372512) and resin was washed three times with PBS. To remove the Fc tag and biotinylate S-2P simultaneously, the protein-bound resin was incubated overnight at 4°C with 200 μg HRV3C [2] and biotinylated using a mixture of 10 μg of BirA enzyme and 100 μM biotin (BirA500 kit, Avidity). After incubation, the protein flowthrough that contains the biotinylated S-2P was collected and further purified by size exclusion chromatography in PBS using a Superose 6 Increase column (Cytiva). The purified, biotinylated S-2P protein was concentrated to 1 mg/ml by centrifugation using an Amicon 50 kDa molecular weight cut-off membrane (Millipore) and aliquots were snap-frozen in liquid nitrogen and stored at -80°C until use.

The RBD cDNA was expressed from the pcDNA3.1(-) vector. The construct encodes from the 5'-end to the 3'-end for (i) the Kozak sequence, (ii) a signal peptide sequence, (iii) aa 328-531 (204 aa) of SARS-CoV-2 S corresponding to the receptor binding domain (RBD), (iv) the 10LnQQ-AVI sequence [1] and (v) an 8X His tag. The RBD cDNA was cloned into the pCDNA3.1(-) vector between the XbaI and BamHI sites to generate the pcDNA3.1-SARS2_RBD_Avi_His vector that was transfected into Expi293 cells following the

manufacturer's instructions. On day 6 post-transfection, cells and cell debris were removed by centrifugation and the supernatant that contained the RBD protein was further clarified through a 0.2 µm PES vacuum filter. The clarified supernatant was then loaded on an HisTrap FF column (Cytiva). The loaded column was washed using a 20 mM HEPES + 250 mM NaCl washing buffer and the RBD protein was eluted using the washing buffer supplemented with 300 mM imidazole. After elution, the RBD protein was diluted to ~3 mg/ml in washing buffer and aliquots were snap frozen in liquid nitrogen and stored at -80°C until further use. Next, aliquots of the RBD protein were biotinylated using the BirA500 kit (Avidity) following the manufacturer's instructions and the biotinylated-RBD was further purified from the excess of biotin by size exclusion chromatography using a Superdex 200-Increase 10/300 GL column in PBS. Aliquots of biotinylated RBD were snap frozen in liquid nitrogen and stored at -80°C until further use.

The biotinylation and antigenicity of the S-2P and RBD probes were confirmed by biolayer interferometry using the Octet RED384 (ForteBio). Assays were performed at 30°C in black 96-well plates (Greiner Bio-One #655209) with shaking. The running buffer used was 10x Kinetics Buffer (ForteBio #18-1105) diluted to 1x with PBS. Streptavidin biosensors (ForteBio) were hydrated in 1x running buffer for at least 10 min prior to beginning the assays. Human anti-S-specific antibodies 2M-10B11[3] and C105 [4] were used to confirm binding to the RBD probe, and 0304-3H3 [3] and C105 were used to confirm binding to the S-2P probe. Antibodies were serially diluted using seven three-fold dilutions from a starting concentration of 100 µg/ml. Streptavidin biosensors were loaded with biotinylated S-2P (1 µg/ml) or RBD (0.4 µg/ml) for 5 min. Biosensors were next washed for 3 min in 1x running buffer, and then dipped into antibody dilutions for 5 min. After antibody binding, biosensors were moved to 1x running buffer for 20

min to monitor dissociation. The background binding observed from a probe-loaded sensor dipped into a control well with no antibody was subtracted from the test wells. The biotinylated S-2P probe efficiently bound to C105 (RBD-specific) and 0304-3H3 (binds outside RBD), while the biotinylated RBD probe efficiently bound to C105 and 2M-10B11 (RBD specific).

Streptavidin (SA)-BV605 (BD Biosciences) and streptavidin (SA)-BV421 (BioLegend) were used to label biotinylated S-2P and RBD, respectively, and were made fresh the day of the experiment. The S-2P probe was mixed to SA-BV605 at a 4:1 molar ratio in 1/3 increments, each with a 20 min incubation, prior to using the conjugated probe for flow cytometry staining. The RBD probe was conjugated to SA-BV421 using the same strategy as described for S-2P. Due to the small molecular weight of the RBD (25,000 Da) the MW of only the SA portion (60,000 Da) of the fluorophore was used to determine the molar ratio. Therefore, the RBD:SA 4:1 molar ratio was used to determine the final quantity of SA-BV421 that was mixed with the RBD following the same protocol than for S-2P. Each conjugated probe was titered using PBMCs isolated from the blood of a healthy donor from the NIH blood bank. The optimal amount of S-2P and RBD probes was determined to be 0.3 μg of each probe per reaction.

Supplementary Methods 2. Antibody-dependent cellular phagocytosis assay (ADCP).

To investigate stimulation of phagocytic activity induced by anti-S antibodies in serum samples of immunized macaques, ADCP assays were performed using a modification of a previously published method [5]. The SARS-CoV-2 S-2P protein (corresponding to the sequence of the ancestral Wuhan-1 strain, kind gift from Dr. Dominic Esposito, Protein Expression Laboratory, Frederick National Laboratory for Cancer Research) was mixed with EZ Link NHS-Biotin (ThermoFischer, cat# 20217; 1 mg of biotin powder dissolved in 295 μl of DMSO) for 2

hours on ice. Excess biotin was removed by adding 15 ml of PBS to the mixture followed by centrifugation at 1,700 x g for 5 min at 4°C using an Amicon 30K MW cut-off membrane (Millipore, cat# UFC903024). This procedure was repeated twice. The last centrifugation was performed until the volume of S-2P protein was between 500 to 700 µl. Then, the purified biotinylated S-2P protein was collected and its concentration was determined by Bradford protein assay.

Forty µl of FluoSpheres Neutravidin-Labelled Microspheres with yellow-green fluorescence (ThermoFisher, cat# F8776) were transferred into a 1.5 ml sterile Eppendorf tube and washed with 1 ml PBS + 0.5% BSA. Then, microspheres were centrifugated at 6,800 x g for 5 min at 4°C and the supernatant was discarded. Microspheres were washed one more time following the same procedure for a total of two washes. After the second wash, beads were resuspended in 200 µl of PBS + 0.5% BSA. Then, 40 µg of purified biotinylated S-2P protein was mixed with the microspheres and the volume was brought to 500 µl using PBS + 0.5% BSA. The bead-S2-P mixture was incubated overnight at 4°C in the dark with rotation. On the following day, conjugated microspheres were washed twice using PBS and once with PBS + 0.5% BSA and centrifugated at 6,800 x g for 5 min at 4°C between each wash. After the third wash, the conjugated microspheres were resuspended in 400 µl of PBS + 0.5% BSA and were stored at 4°C in the dark for up to one month.

Serum samples from immunized macaques were added to U-bottom 96 well plates (Costar, cat# 3799) at a starting dilution of 1:10 and then were three-fold serially diluted in PBS + 0.5% BSA for a total of 12 dilutions. One µl of the stock of S-conjugated beads was added to each well of the serum dilutions and plates were incubated for two hours at 37°C. Next, $2x10^4$ THP-1 cells were added to each well and plates were incubated at 37°C for another two

hours. After incubation, plates were washed twice with PBS and centrifugated at 150 x g for 5 min at RT. After washing, cells were fixed with 2% PFA final in PBS + 0.5% BSA. Plates were kept at 4°C in the dark until analysis of FITC signal by flow cytometry on the BD Canto II. The frequency of FITC positive THP-1 cells for each dilution series was used to calculate IC_{50} values. Serum from one of the MPV/S-2P immunized macaques on day 21 post-boost was included on each assay plate as a positive control sample, and the reported IC_{50} values were normalized to the average of the positive control values from all analyzed plates.

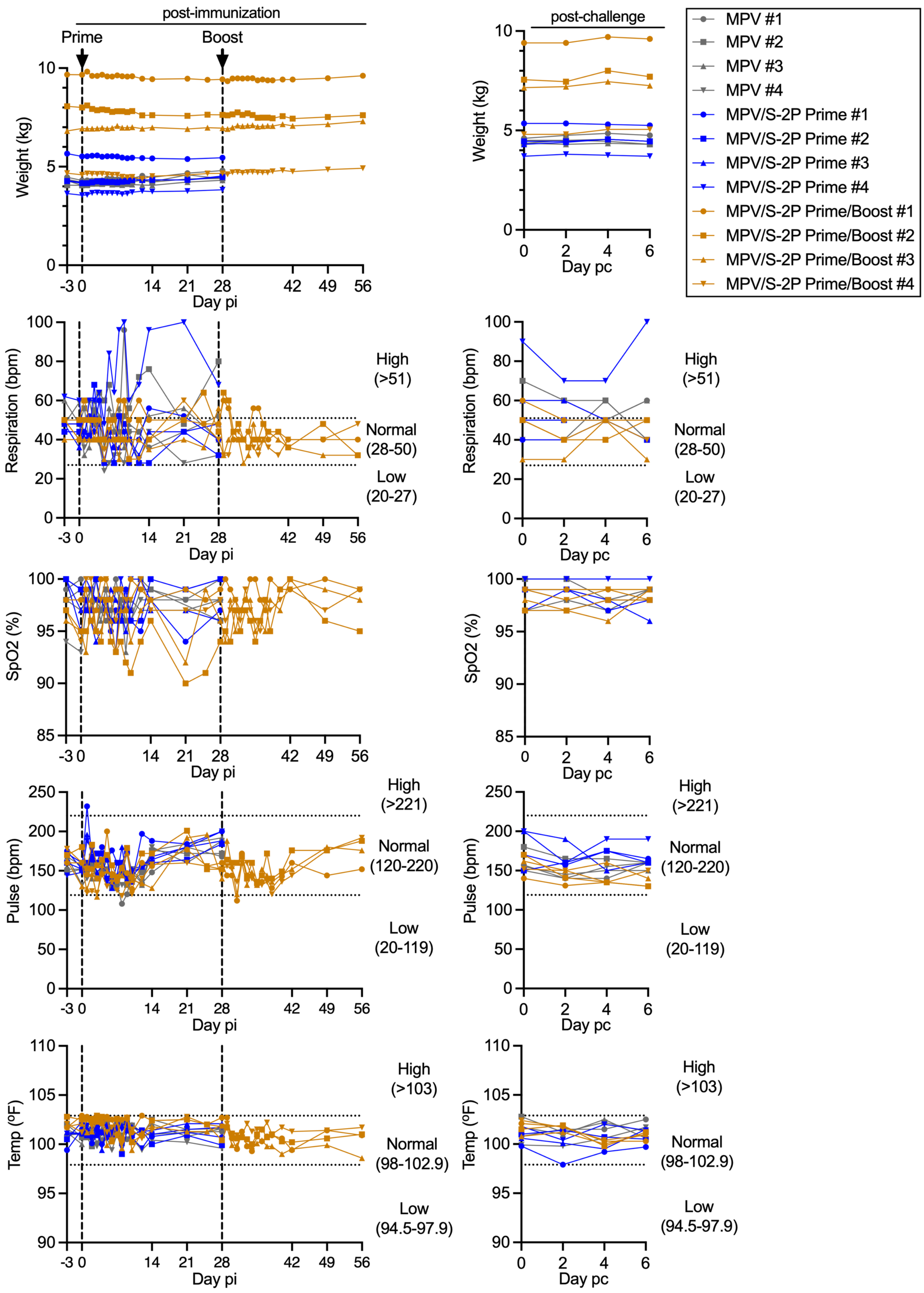

post-immunization
Prime
Boost
Weight (kg)
Day pi
post-challenge
Day pc
MPV #1
MPV #2
MPV #3
MPV #4
MPV/S-2P Prime #1
MPV/S-2P Prime #2
MPV/S-2P Prime #3
MPV/S-2P Prime #4
MPV/S-2P Prime/Boost #1
MPV/S-2P Prime/Boost #2
MPV/S-2P Prime/Boost #3
MPV/S-2P Prime/Boost #4
Respiration (bpm)
High (>51)
Normal (28-50)
Low (20-27)
SpO2 (%)
Pulse (bpm)
High (>221)
Normal (120-220)
Low (20-119)
Temp (ºF)
High (>103)
Normal (98-102.9)
Low (94.5-97.9)

Supplementary Fig. 1, related to Fig. 1. Vitals signs of macaques.

Animals were immunized as shown in Fig. 1A. Individual animals that were primed with empty MPV vector (n=4) are shown as grey symbols, animals primed with MPV/S-2P (n=4) are in blue, and animals primed and boosted with MPV/S-2P (n=4) are in gold. Weight, respiration rate, saturation of peripheral oxygen (SpO_2), heart rate, and rectal temperature were monitored on the indicated day post-immunization (pi) or post-challenge (pc). Beats per minute, bpm. Source data are provided in the Source Data file.

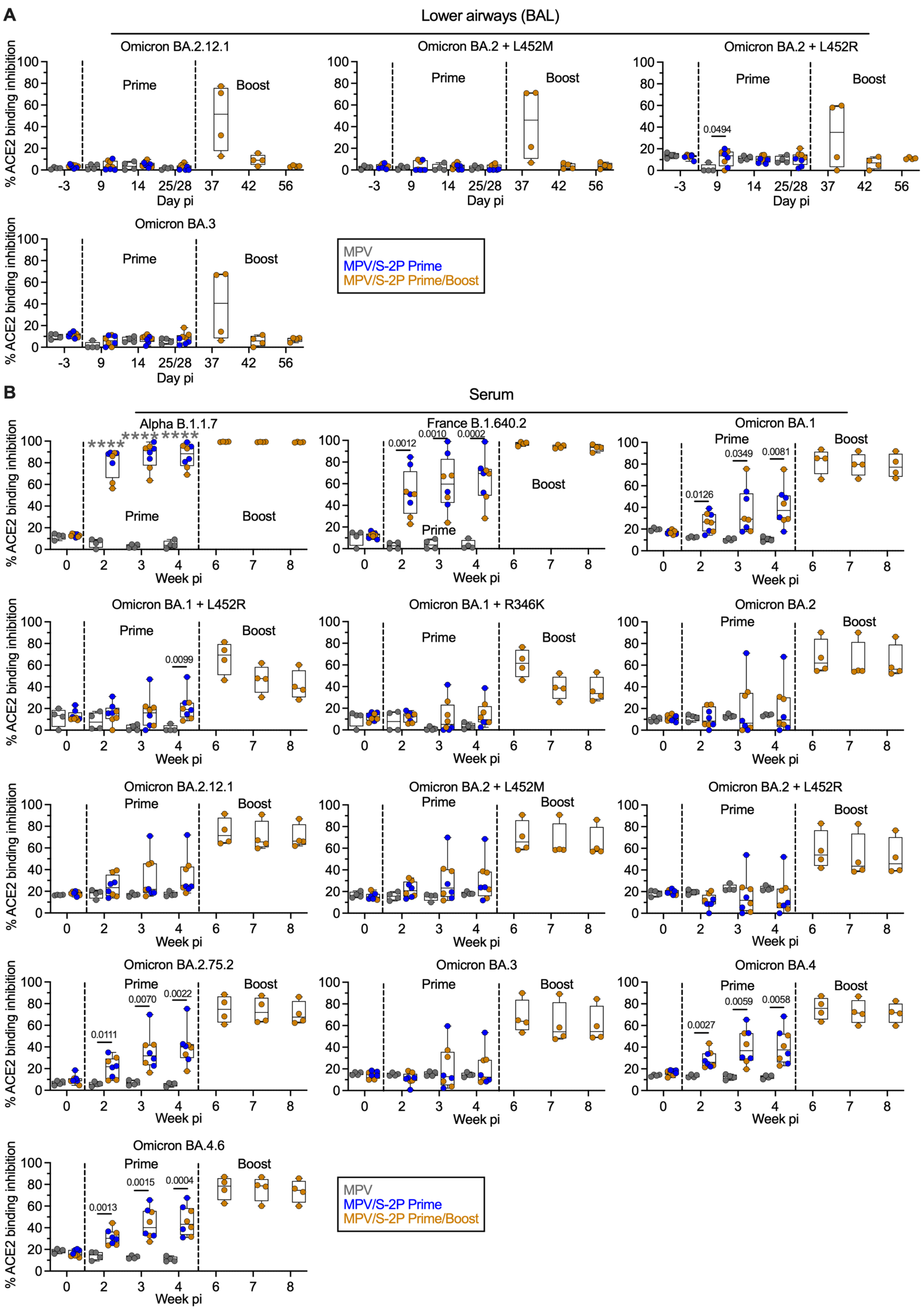
A
Lower airways (BAL)
Omicron BA.2.12.1
Omicron BA.2 + L452M
Omicron BA.2 + L452R
Omicron BA.3
% ACE2 binding inhibition
Prime
Boost
0.0494
-3
9
14
25/28
37
42
56
Day pi
MPV
MPV/S-2P Prime
MPV/S-2P Prime/Boost
B
Serum
Alpha B.1.1.7
France B.1.640.2
Omicron BA.1
Omicron BA.1 + L452R
Omicron BA.1 + R346K
Omicron BA.2
Omicron BA.2.12.1
Omicron BA.2 + L452M
Omicron BA.2 + L452R
Omicron BA.2.75.2
Omicron BA.3
Omicron BA.4
Omicron BA.4.6
****
0.0012
0.0010
0.0002
0.0126
0.0349
0.0081
0.0099
0.0111
0.0070
0.0022
0.0027
0.0059
0.0058
0.0013
0.0015
0.0004
Week pi
MPV
MPV/S-2P Prime
MPV/S-2P Prime/Boost

Supplementary Fig. S2, related to Fig. 2G and 3E. Inhibition of ACE2 binding to S by mucosal and serum samples from MPV/S-2P immunized macaques.

Bronchoalveolar lavages (BAL) (**A**) and serum samples (**B**) from MPV and MPV/S-2P immunized macaques were harvested on the indicated day or week pi. The relative inhibition of binding of soluble, tagged angiotensin converting enzyme receptor (ACE2) to purified SARS-CoV-2 S proteins from the indicated Variants of concern by the BAL (A) or serum (**B**) samples was evaluated and expressed as % ACE2 binding inhibition relative a no-serum control. Individual animals that were primed with empty MPV vector (n=4) are shown as grey symbols, animals primed with MPV/S-2P (n=4) are in blue, and animals primed and boosted with MPV/S-2P (n=4) are in gold. The medians (lines), min and max values (whiskers), 25th to 75th quartiles (boxes), and individual value are shown for MPV primed (n=4), MPV/S-2P primed (n=8) and boosted (n=4) macaques. Two-way ANOVA with Sidak post-test; exact p values are indicated for levels of significance $p<0.05$ unless $p<0.0001$ (****). Source data are provided in the Source Data file.

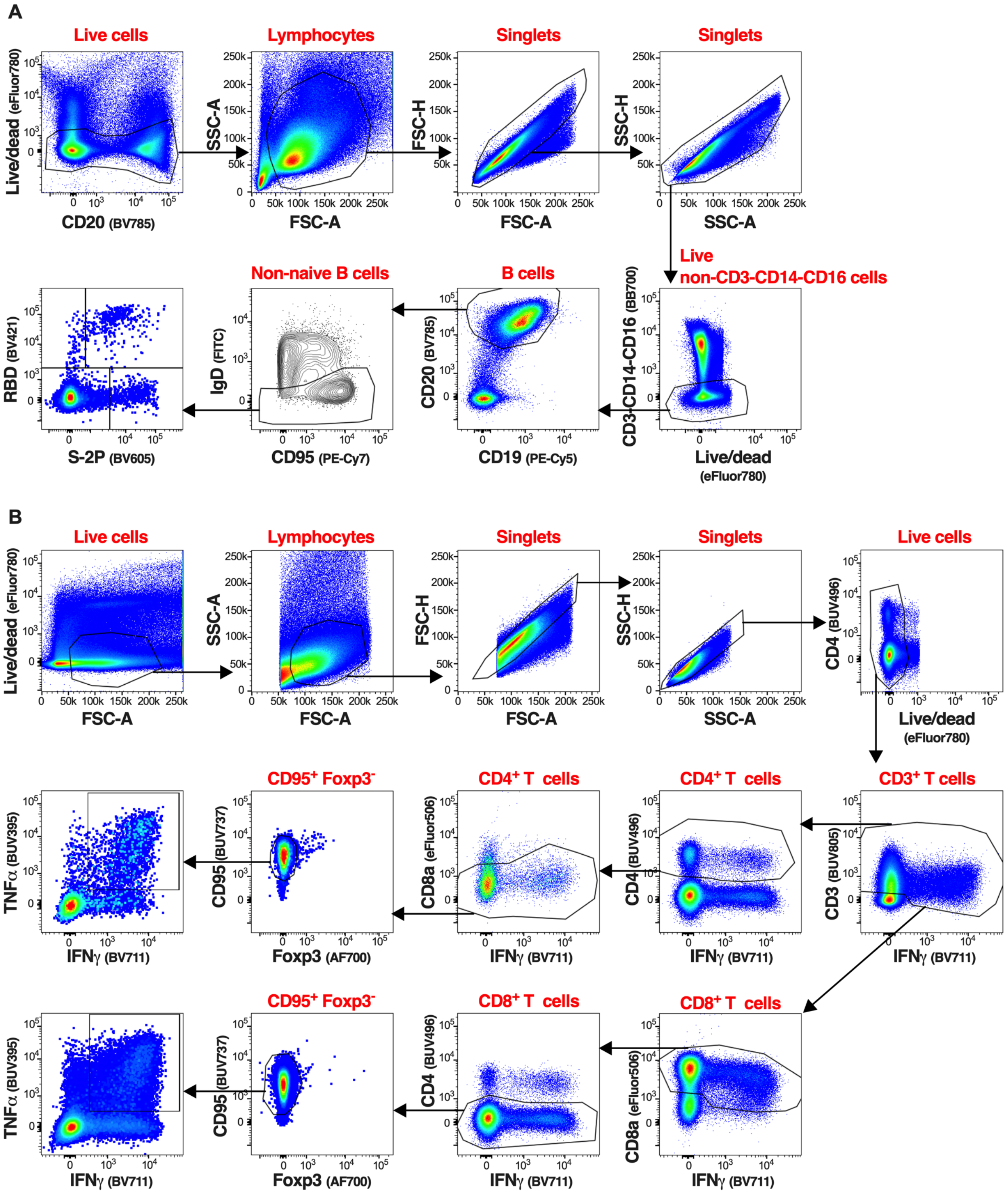
A
Live cells
Lymphocytes
Singlets
Singlets
Live/dead (eFluor780)
CD20 (BV785)
SSC-A
FSC-A
FSC-H
SSC-H
Live
non-CD3-CD14-CD16 cells
Non-naive B cells
B cells
RBD (BV421)
S-2P (BV605)
IgD (FITC)
CD95 (PE-Cy7)
CD20 (BV785)
CD19 (PE-Cy5)
CD3-CD14-CD16 (BB700)
Live/dead
(eFluor780)
B
Live cells
Lymphocytes
Singlets
Singlets
Live cells
Live/dead (eFluor780)
FSC-A
SSC-A
FSC-H
SSC-H
SSC-A
CD4 (BUV496)
Live/dead
(eFluor780)
CD95⁺ Foxp3⁻
CD4⁺ T cells
CD4⁺ T cells
CD3⁺ T cells
TNFα (BUV395)
IFNγ (BV711)
CD95 (BUV737)
Foxp3 (AF700)
CD8a (eFluor506)
CD4 (BUV496)
CD3 (BUV805)
CD95⁺ Foxp3⁻
CD8⁺ T cells
CD8⁺ T cells

Supplementary Fig. S3, related to Fig. 4 to 6. Gating strategy used to identify spike-specific B-ells and spike-specific $CD4^+$ and $CD8^+$ T-cells from the blood or bronchoalveolar lavage of immunized macaques.

Representative flow cytometry dot plots of cells isolated from PBMC that describe the gating strategy to identify the spike (S)-specific B-cell populations described in Fig. 3. Live cells were gated using live/dead staining and the B-cell marker CD20. Lymphocytes were identified based on forward- and side-scatter areas. Next, singlets were identified with a first gate based on forward-scatter height and forward-scatter area followed by a second gate based on side-scatter height and side-scatter area. An additional live/dead gate was used to exclude remaining dead cells and $CD3^+$, $CD14^+$, and $CD16^+$ cells. Live single B-cells identified using CD19 and CD20 and naïve (IgD^+) B-cells were removed from analysis. S-specific B-cells were identified in the live, single, $CD3^-$, $CD14^-$ $CD16^-$ $CD19^+$ $CD20^+$ IgD^- $CD95^{+/-}$ B-cell population. Then, tagged SARS-CoV-2 receptor binding domain (RBD) and S-2P protein probes were used to identify RBD and/or S-2P-binding B-cells, which were used for additional phenotypic analysis described in Fig. 4. The phenotypic analyses in Fig. 4 were done on live, single, non-naïve (IgD^-) $CD95^{+/-}$ B-cells. (**B**) Representative flow cytometry dot plots of cells isolated from a BAL sample, describing the typical gating strategy used to identify the $CD4^+$ and $CD8^+$ T-cell populations described in Fig. 5 and 6. The same gating strategy was applied to identify and analyze the $CD4^+$ and $CD8^+$ T-cells from PBMC isolated from the blood and after SARS-CoV-2 challenge (Fig. S4 and S6, respectively). Live cells were selected as in (A). The live single $CD3^+$ $IFNg^+$ T-cells were next gated using CD3 and interferon gamma (IFNg). As CD3 expression can be downregulated on activated T-cells, a wide CD3 gate was applied. $IFNg^+$ $CD4^+$ or $IFNg^+$ $CD8^+$ T-cells were next identified and CD8 or CD4 gating was performed to remove potentially

remaining $CD8^+$ or $CD4^+$ T-cells from the $CD4^+$ or $CD8^+$ T-cell populations, respectively. Finally, non-naïve, non-regulatory $CD4^+$ or $CD8^+$ T-cells were identified using CD95 and Foxp3, respectively. The phenotypic analyses described in Fig. 5, 6, S4, and S6 were performed on live single $CD3^+$ $CD4^+$ $CD8^-$ $CD95^+$ $Foxp3^-$ or live single $CD3^+$ $CD8^+$ $CD4^-$ $CD95^+$ $Foxp3^-$ T-cells. TNFa, tumor necrosis factor alpha.

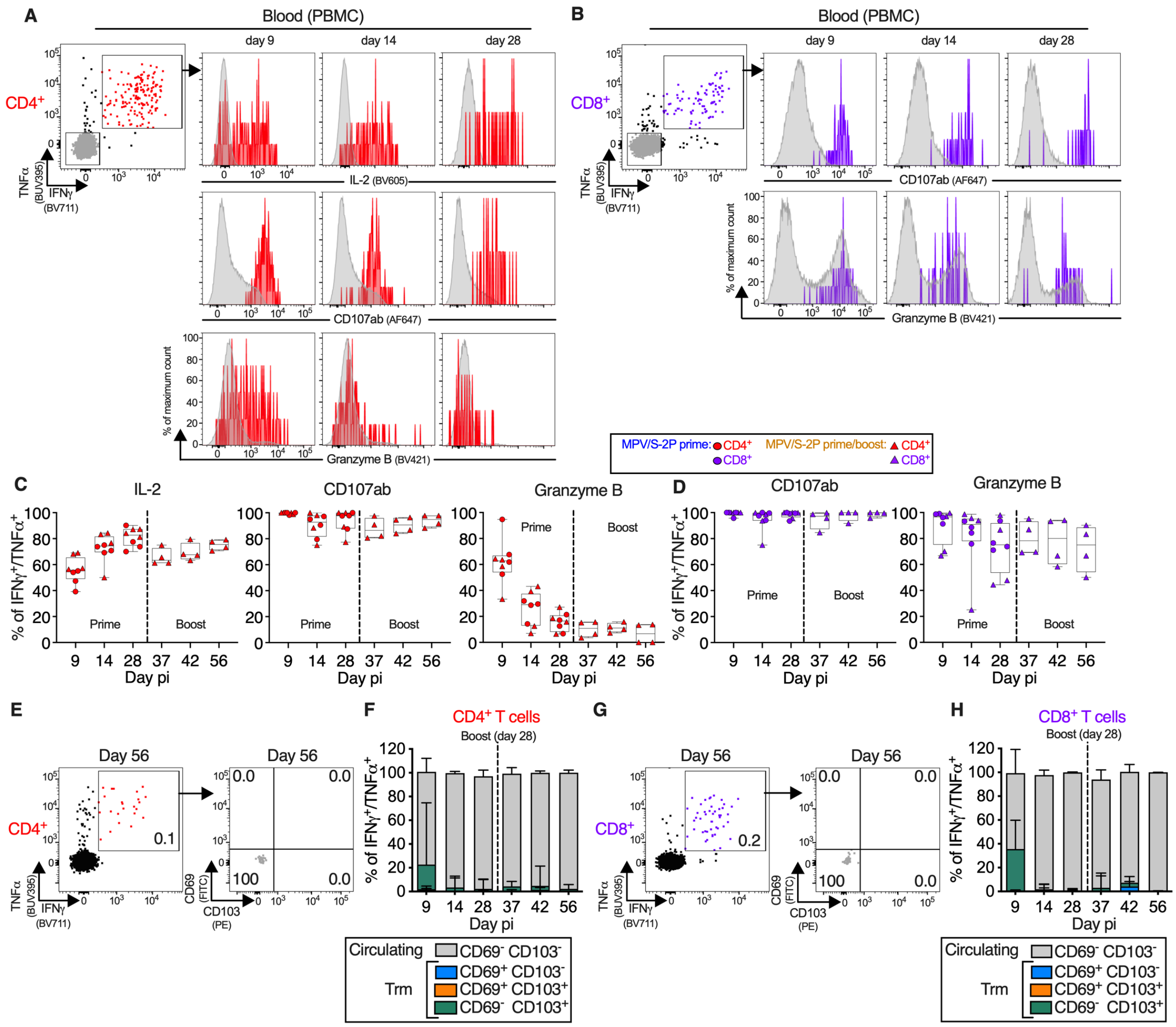

Supplementary Fig. S4, related to Fig. 5 and 6. Phenotype of SARS-CoV-2 S-specific CD4$^+$ and CD8$^+$ T-cells in the blood of the MPV/S-2P immunized macaques.

Three groups of four macaques each were immunized with MPV or one or two doses of MPV/S-2P as outlined in Fig. 1A. (**A, B**) Dot blots of the CD4$^+$ (**A**) or CD8$^+$ (**B**) T-cells from the blood of representative MPV/S-2P-immunized macaques showing the gating of S-specific interferon gamma/tumor necrosis factor alpha positive (IFNγ$^+$/TNFα$^+$) CD4$^+$ (red, **A**) or CD8$^+$ (purple, **B**) T-cells. The levels of expression of interleukin 2 (IL-2) (for CD4$^+$ T-cells only), CD107ab and

granzyme B by the IFNγ$^+$/TNFα$^+$ CD4$^+$ or CD8$^+$ T-cells from representative macaques are shown as histograms on the indicated day pi with the IFNγ$^+$/TNFα$^+$ CD4$^+$ or CD8$^+$ T-cells (grey) used for comparison. (**C-D**) % of IFNγ$^+$/TNFα$^+$ CD4$^+$ (red, **C**) or CD8$^+$ (purple, **D**) T-cells in the blood of the MPV/S-2P-immunized macaques that expressed IL-2 (for CD4$^+$ T-cells only), CD107ab or granzyme B on the indicated day pi. Dotted lines indicate the boost with MPV/S-2P, 28 days after the first immunization. MPV/S-2P-primed and MPV/S-2P-primed/boosted macaques are represented by circles or triangles, respectively. The medians (lines), min and max values (whiskers), 25th to 75th quartile, and individual value are shown for MPV/S-2P primed (n=8) and boosted (n=4) macaques. (**E-H**) Lack of expression of tissue resident memory (Trm) markers by S-specific CD4$^+$ and CD8$^+$ T-cells from blood. (**E**, **G**) Representative dot plots showing the gating on S-specific IFNγ$^+$/TNFα$^+$ CD4$^+$ (**E**) or CD8$^+$ (**G**) T-cells (left panels). CD69 and CD103 were used to differentiate circulating (CD69$^-$ CD103$^-$, grey) and Trm [CD69$^+$ CD103$^-$ (blue), CD69$^+$ CD103$^+$ (orange) and CD69$^-$ CD103$^+$ (green)] S-specific IFNγ$^+$/TNFα$^+$ T-cells isolated from blood (right panels, % indicated). (**F**, **H**) The median % of the circulating and each of the three Trm S-specific IFNγ$^+$/TNFα$^+$ CD4$^+$ (**F**) or CD8$^+$ (**H**) T-cell subsets present in blood of MPV/S-2P-immunized macaques on indicated days are stacked (median and range are shown). N = eight MPV/S-2P-primed macaques and n = four MPV/S-2P-boosted macaques. Source data are provided in the Source Data file.

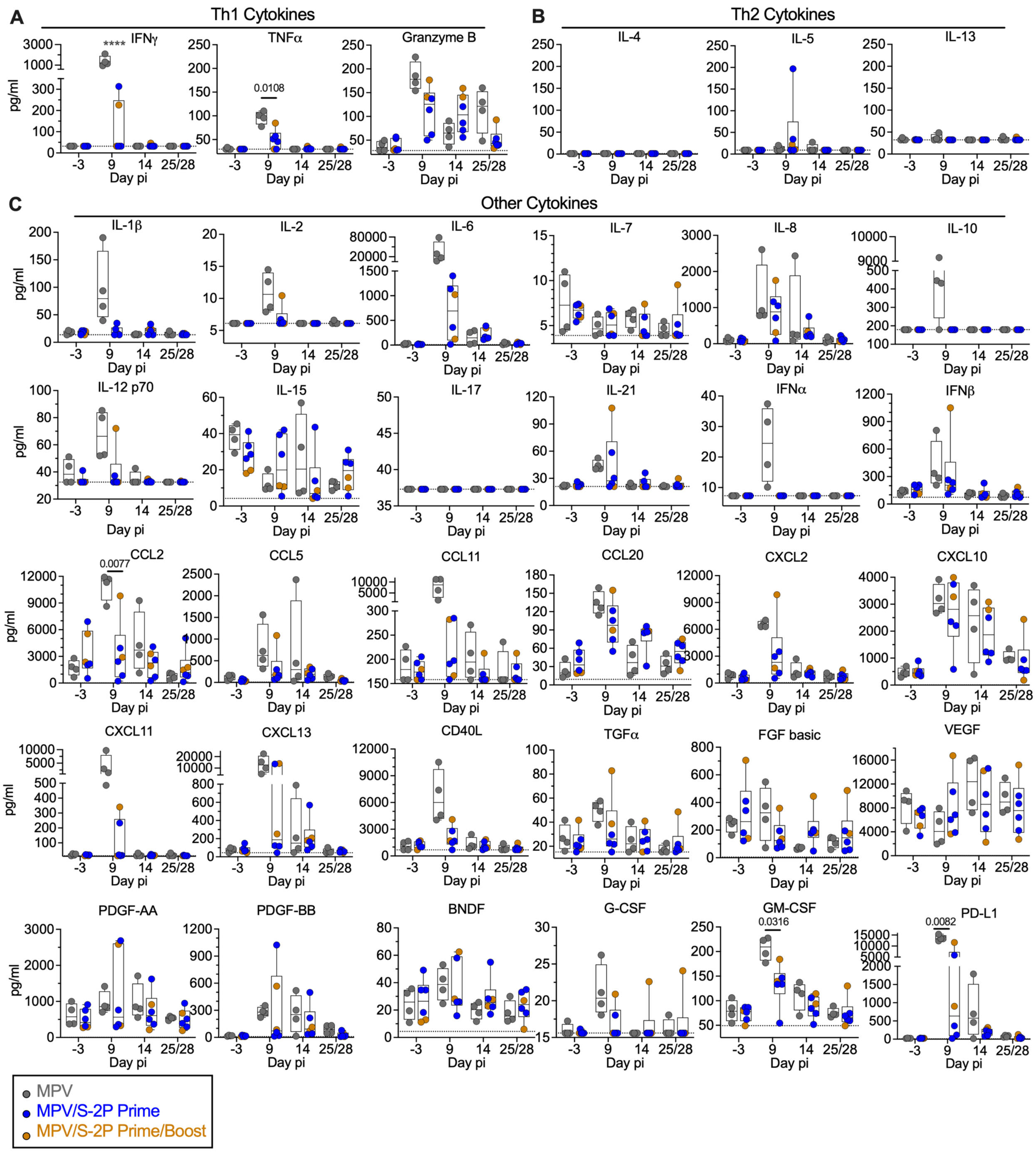

Supplementary Fig. S5, related to Fig. 5 and 6. Cytokine concentration in bronchoalveolar lavage fluid of MPV/S-2P immunized macaques.

Three groups of four macaques each were immunized with MPV or MPV/S-2P as outlined in Fig. 1A. Four MPV-immunized macaques (grey) and six of eight MPV/S-2P immunized

macaques with the strongest T-cell responses in the BAL were chosen for analysis of the post-immunization cytokine response in the lower airways following priming (responses after the boost were not analyzed). Bronchoalveolar lavage (BAL) fluid collected on the indicated days after immunization with MPV or a single dose of MPV/S-2P was concentrated tenfold using Amicon concentration tubes (3 kDa cutoff, Millipore), and the concentrations of 36 cytokines were evaluated in duplicate by a multiplex bead-based immunoassay (NHP XL Cytokine Luminex Performance Premixed Kit; Bio-Techne Corporation, cat# FCSTM21) following the manufacturer's instructions. Data were acquired on Millipore MagPIX (Millipore) and analyzed using the Luminex xPONENT 4.3 software. The concentration of each cytokine was expressed in pg/ml. Th-1 related cytokines (**A**), Th-2 related cytokines (**B**), and other cytokines (**C**) are shown. The median (lines), min and max values (whiskers), 25th to 75th quartiles (boxes), and individual value are shown for MPV primed (n=4), MPV/S-2P primed (n=8) and boosted (n=4) macaques. Two-way ANOVA with Sidak post-test; exact p values are indicated for levels of significance $p<0.05$. Source data are provided in the Source Data file.

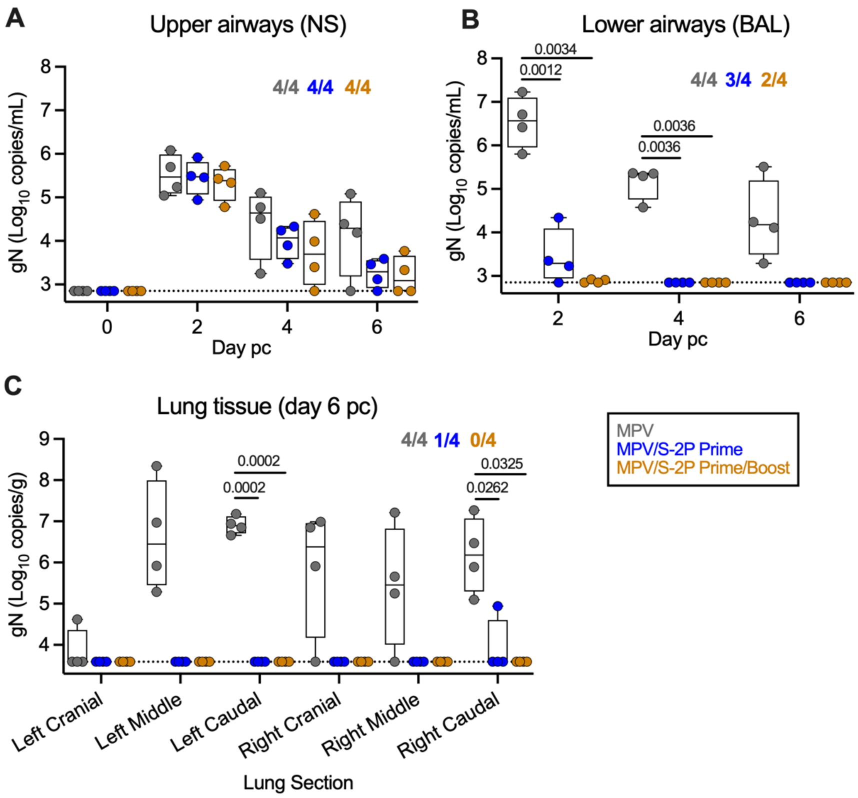

Supplementary Fig. S6, related to Fig. 7. No detectable SARS-CoV-2 challenge virus replication in macaques that received a prime/boost regimen of MPV/S-2P.

On days 31 or 32 post-prime [MPV- (grey) and MPV/S-2P-immunized macaques, blue] or on day 58 (corresponding to day 30 post-boost; MPV/S-2P-immunized macaques, gold, n=4), macaques were challenged IN/IT with 6.3 $\log_{10}$ $TCID_{50}$ of SARS-CoV-2, strain WA1/2020 (Fig. 1A). (**A**-**B**) SARS-CoV-2 genomic RNA in the upper (**A**) and lower airways (**B**) following challenge. Nasopharyngeal swabs (NS) and bronchoalveolar lavages (BAL) were collected, and SARS-CoV-2 genomic N (gN) RNA, indicative of the presence of SARS-CoV-2 RNA, was quantified by RT-qPCR. The gN assay also detects RNA from input challenge virus. (**C**) SARS-CoV-2 quantification from lung tissues. On day 6 pc, SARS-CoV-2 gN RNA was quantified by RT-qPCR in six regions of lung tissues from each macaque. The number of macaques with detectable gN RNA is indicated. Limit of detection: 2.85 $\log_{10}$ copies/ml for NS and BAL; 3.6

log_{10} copies/g for lung tissue. (**A-C**) The medians (lines), min and max values (whiskers), 25th to 75th quartiles (boxes), and individual value are shown for MPV primed (n=4), MPV/S-2P primed (n=8) and boosted (n=4) macaques. Two-way ANOVA with Sidak post-test; exact p values are indicated for levels of significance $p<0.05$. Source data are provided in the Source Data file.

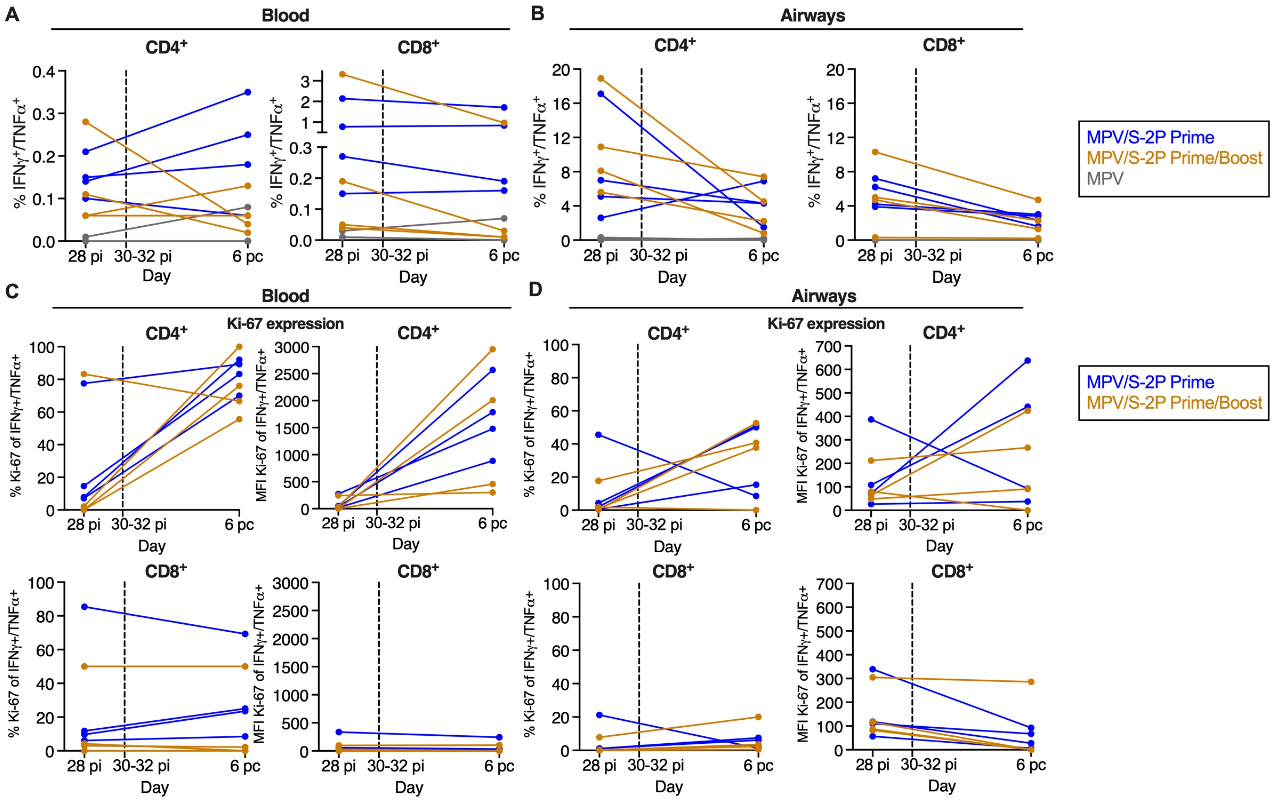

Supplementary Fig. S7, related to Fig. 5. Phenotype of S-specific IFNγ$^+$/TNFα$^+$ CD4$^+$ and CD8$^+$ T-cells in the blood and lower airways of immunized macaques before and after SARS-CoV-2 challenge.

Three groups of four macaques each were immunized with MPV or MPV/S-2P as outlined in Fig. 1A. (**A**, **B**) Background-corrected frequencies of S-specific IFNg$^+$/TNFa$^+$ CD4$^+$ or CD8$^+$ T-cells from blood (**A**) or airways (**B**) on day 28 pi and on day 6 post-challenge [performed on day 31/32 (groups 1 and 2, n=4 per group) or 58 pi (group 3, n=4)]. These frequencies are similar to those shown in Fig. 5B-C, and Fig. 5E-F for the blood and airways, respectively. (**C, D**) % and median fluorescence intensity (MFI) of proliferation marker Ki-67 by IFNg$^+$/TNFa$^+$ CD4$^+$ (top panels) or CD8$^+$ (bottom panels) T-cells from blood (**C**) or airways (**D**). Source data are provided in the Source Data file.

Supplementary References

1 Zhou, T. *et al.* Structure-Based Design with Tag-Based Purification and In-Process Biotinylation Enable Streamlined Development of SARS-CoV-2 Spike Molecular Probes. *Cell Rep* **33**, 108322, doi:10.1016/j.celrep.2020.108322 (2020).

2 Antoniou, G., Papakyriacou, I. & Papaneophytou, C. Optimization of Soluble Expression and Purification of Recombinant Human Rhinovirus Type-14 3C Protease Using Statistically Designed Experiments: Isolation and Characterization of the Enzyme. *Mol Biotechnol* **59**, 407-424, doi:10.1007/s12033-017-0032-9 (2017).

3 Chi, X. *et al.* A neutralizing human antibody binds to the N-terminal domain of the Spike protein of SARS-CoV-2. *Science* **369**, 650-655, doi:10.1126/science.abc6952 (2020).

4 Barnes, C. O. *et al.* Structures of Human Antibodies Bound to SARS-CoV-2 Spike Reveal Common Epitopes and Recurrent Features of Antibodies. *Cell* **182**, 828-842 e816, doi:10.1016/j.cell.2020.06.025 (2020).

5 Ackerman, M. E. *et al.* A robust, high-throughput assay to determine the phagocytic activity of clinical antibody samples. *J Immunol Methods* **366**, 8-19, doi:10.1016/j.jim.2010.12.016 (2011).
