# Supplementary material for: Mucosal prime-boost immunization with live murine pneumonia virus-vectored SARS-CoV-2 vaccine is protective in macaques
Source: Nat Commun. 2024 Apr 26;15:3553. doi: 10.1038/s41467-024-47784-6 (PMC11053155; doi:10.1038/s41467-024-47784-6)
Supplement: Supplementary file 3 — Reporting Summary [file 41467_2024_47784_MOESM3_ESM.pdf]

nature portfolio

Corresponding author(s): Ursula Buchholz
Cyril Le Nouen

Last updated by author(s): 04/10/2024

Reporting Summary

Nature Portfolio wishes to improve the reproducibility of the work that we publish. This form provides structure for consistency and transparency in reporting. For further information on Nature Portfolio policies, see our Editorial Policies and the Editorial Policy Checklist.

Statistics

For all statistical analyses, confirm that the following items are present in the figure legend, table legend, main text, or Methods section.

n/a	Confirmed	
☐	☒	The exact sample size (*n*) for each experimental group/condition, given as a discrete number and unit of measurement
☐	☒	A statement on whether measurements were taken from distinct samples or whether the same sample was measured repeatedly
☐	☒	The statistical test(s) used AND whether they are one- or two-sided<br>*Only common tests should be described solely by name; describe more complex techniques in the Methods section.*
☐	☒	A description of all covariates tested
☐	☒	A description of any assumptions or corrections, such as tests of normality and adjustment for multiple comparisons
☐	☒	A full description of the statistical parameters including central tendency (e.g. means) or other basic estimates (e.g. regression coefficient) AND variation (e.g. standard deviation) or associated estimates of uncertainty (e.g. confidence intervals)
☐	☒	For null hypothesis testing, the test statistic (e.g. *F*, *t*, *r*) with confidence intervals, effect sizes, degrees of freedom and *P* value noted<br>*Give P values as exact values whenever suitable.*
☒	☐	For Bayesian analysis, information on the choice of priors and Markov chain Monte Carlo settings
☒	☐	For hierarchical and complex designs, identification of the appropriate level for tests and full reporting of outcomes
☒	☐	Estimates of effect sizes (e.g. Cohen's *d*, Pearson's *r*), indicating how they were calculated

Our web collection on statistics for biologists contains articles on many of the points above.

Software and code

Policy information about availability of computer code

Data collection	Synergy Neo2 (BioTek)<br>MESO Quickplex SQ 120 mm (Meso Scale)<br>BD FACSymphony A5 (BD Biosciences)<br>QuantStudio 7 PRO (Thermo Fisher)<br>LI_COR Odyssey CLX (LI-COR Biosciences)<br>Millipore MagPIX (Millipore
Data analysis	GraphPad Prism versions 9.0 and 9.5<br>Methodical Mind 1.0.38 (Meso Scale)<br>Flowjo version 10<br>QuantStudio 6/7 Pro Touchscreen instrument Operating Software<br>Standard Curve on https://apps.thermofisher.com<br>ImageStudioLite 5.2.5 (LI-COR Biosciences)<br>Luminex xPONENT 4.3

For manuscripts utilizing custom algorithms or software that are central to the research but not yet described in published literature, software must be made available to editors and reviewers. We strongly encourage code deposition in a community repository (e.g. GitHub). See the Nature Portfolio guidelines for submitting code & software for further information.

Data

Policy information about availability of data

All manuscripts must include a data availability statement. This statement should provide the following information, where applicable:

- Accession codes, unique identifiers, or web links for publicly available datasets
- A description of any restrictions on data availability
- For clinical datasets or third party data, please ensure that the statement adheres to our policy

The experimental data generated in this study are provided in the main text or in the Supplementary Information/Source Data File.

Nucleotide sequences cited in this manuscript include:
GenBank MN985325 [https://www.ncbi.nlm.nih.gov/nuccore/MN985325]; GISAID accession ID: EPI_ISL_404895 [https://www.epicov.org/epi3/frontend#3f2c48] (SARS-CoV-2 USA-WA1/2020).
GISAID: EPI_ISL_751801 [https://www.epicov.org/epi3/frontend#42f6ed] (SARS-CoV-2 USA/CA_CDC_5574/2020 isolate (lineage B.1.1.7)).
GISAID: EPI_ISL_890360 [https://www.epicov.org/epi3/frontend#2a5858] (SARS-CoV-2 USA/MD-HP01542/2021 isolate (lineage B.1.351).
GenBank AY729016 [https://www.ncbi.nlm.nih.gov/nuccore/58610194] (Murine pneumonia virus).
GenBank MN908947 [https://www.ncbi.nlm.nih.gov/nuccore/MN908947] (SARS-CoV-2, isolate Wuhan Hu-1).

Research involving human participants, their data, or biological material

Policy information about studies with human participants or human data. See also policy information about sex, gender (identity/presentation), and sexual orientation and race, ethnicity and racism.

Reporting on sex and gender	NA
Reporting on race, ethnicity, or other socially relevant groupings	NA
Population characteristics	NA
Recruitment	NA
Ethics oversight	NA

Note that full information on the approval of the study protocol must also be provided in the manuscript.

Field-specific reporting

Please select the one below that is the best fit for your research. If you are not sure, read the appropriate sections before making your selection.

☒ Life sciences ☐ Behavioural & social sciences ☐ Ecological, evolutionary & environmental sciences

For a reference copy of the document with all sections, see nature.com/documents/nr-reporting-summary-flat.pdf

Life sciences study design

All studies must disclose on these points even when the disclosure is negative.

Sample size	Sample size calculations were not performed. Twelve juvenile and young adult male Indian-origin rhesus macaques (Macaca mulatta), seronegative for SARS-CoV-2, were included in this study. We used four rhesus macaques per group, consistent with prior studies of mucosal vaccines in nonhuman primates (for example Le Nouen et al., Cell 185, 4811-25, 2022). Animals were randomly assigned to treatment groups of 4 animals each. The study included sequential time points to evaluate vaccine replication. For analysis of vaccine shedding and immunogenicity after the first dose, data from animals from the prime and prime/boost groups were combined.
Data exclusions	No data were excluded from the analyses.
Replication	This is a nonhuman primate study. Per ACUC requirements, duplicative studies are discouraged. We evaluated 4 animals per group.
Randomization	Animals were randomly assigned to groups 2 and 3.
Blinding	The animal study was performed unblinded due to biosafety and animal facility requirements. While formal blinding was not performed in this study, analyses of animal study samples were performed in a blinded manner whenever possible. Statistics were not calculated until the study was complete and all data had been verified to be accurate.

Reporting for specific materials, systems and methods

We require information from authors about some types of materials, experimental systems and methods used in many studies. Here, indicate whether each material, system or method listed is relevant to your study. If you are not sure if a list item applies to your research, read the appropriate section before selecting a response.

Materials & experimental systems

n/a	Involved in the study
☐	☒ Antibodies
☐	☒ Eukaryotic cell lines
☒	☐ Palaeontology and archaeology
☐	☒ Animals and other organisms
☒	☐ Clinical data
☒	☐ Dual use research of concern
☒	☐ Plants

Methods

n/a	Involved in the study
☒	☐ ChIP-seq
☐	☒ Flow cytometry
☒	☐ MRI-based neuroimaging

Antibodies

Antibodies used

B-cell studies:
Anti-human IgD-FITC, Southern Biotech, Cat # 2030-02, RRID: AB_2795624
Anti-human CD3-BB700, clone SP34-2 BD Biosciences Cat #566518; RRID: AB_2744378
Anti-human CD16-BB700, clone 3G8 BD Biosciences Cat #746199; RRID: AB_2743545
Anti-human CD14-BB700, clone M5E2, BD Biosciences Cat # 745790; RRID: AB_2743246
Anti-human IgM-BV510, clone G20-127, BD Bioscience Cat # 563113; RRID: AB_2738010
Anti-human CD27-BV711, clone O323, Biolegend Cat # 302834; RRID: AB_2563809
Anti-human CD20-BV785, clone 2H7, Biolegend Cat # 302356; RRID: AB_2566316
Anti-human CD21-BUV395, clone B-ly4, BD Biosciences Cat # 740288; RRID: AB_2740027
Anti-human IgG-BUV737, clone G18-145, BD Biosciences Cat # 612819; RRID: AB_2870143
Anti-human CD138-APC, clone MI15, Biolegend Cat # 356506; RRID: AB_2561880
Anti-rhesus CD38-PE, clone OKT10, Caprico Biotechnologies Cat # 100826
Anti-human IgA-TxRD, Southern Biotech, Cat # 2050-07, ; RRID: AB_2795705
Anti-human CD19-PE-Cy5, clone J3-119, Beckman Cat # IM2643U, ; RRID: AB_131160
Anti-human CD95-PE-Cy7, clone DX2, Biolegend Cat # 305622; RRID: AB_2100369

T-cell studies:
Anti-human CD69-FITC, Clone FN50 Biolegend Cat #310903; RRID: AB_314838
Anti-human Granzyme B-BV421, Clone GB11 BD Biosciences Cat #563389; RRID: AB_2738175
Anti-human CD8a-eFluor 506, Clone RPA-T8 Thermo Fisher Cat #69-0088-42; RRID: AB_2637468
Anti-human IL-2-BV605, Clone MQ1-17H12 Biolegend Cat #500332; RRID: AB_2563877
Anti-human IFNg-BV711, Clone 4S.B3 Biolegend Cat #502540; RRID: AB_2563506
Anti-human IL-17-BV785, Clone BL168 Biolegend Cat #512338; RRID: AB_2566765
Anti-human TNFa-BUV395, Clone Mab11 BD Biosciences Cat #563996; RRID: AB_2738533
Anti-human CD4-BUV496, Clone SK3 BD Biosciences Cat #612937, RRID: AB_2916881
Anti-human CD95-BUV737, Clone DX2 BD Biosciences Cat #612790, RRID: AB_2870117
Anti-human CD3-BB700, Clone SP34-2 BD Biosciences Cat #566518; RRID: AB_2744378
Anti-human CD107a-AF647, Clone H4A3 Biolegend Cat #328612; RRID: AB_1227506
Anti-human CD107b-AF647, Clone H4B4 Biolegend Cat #354312; RRID: AB_2721405
Anti-human CD103-PE, Clone B-Ly7 eBioscience Cat #12-1038-42; RRID: AB_11150242
Anti-human CD28-PE/Dazzle 594, Clone CD28.2 Biolegend Cat #302942; RRID: AB_2564235
Anti-human Ki-67-PE-Cy7, Clone B56 BD Biosciences Cat #561283; RRID: AB_10716060
Anti-human Foxp3-AF700, Clone PCH101 Thermo Fisher Cat #56-4776-41; RRID: AB_1582210
Anti-monkey IgG(H+L)-HRP Thermo Fisher Cat #PA1-84631; RRID: AB_933605
Anti-monkey IgA(alpha chain)-biotin Alpha Diagnostic International Cat #70049

Anti-rhesus J chain-biotin NHPRR Cat# PR-3316; RRID: AB_2819359
Anti-monkey IgM-biotin Brookwood Biomedical Cat #1152
Rabbit hyperimmune serum against MPV virions (Brock LG, et al.; J Virol 92, (2018) PMID: 29925656).
Goat hyperimmune serum against SARS-CoV-2 S-2P [Liu X, et al.;. Proc Natl Acad Sci U S A 118, (2021); PMID: 34876520).
Human monoclonal antibody CR3022 [Park HS, et al.; NPJ Vaccines 7, 72 (2022); PMID:35764659]
Anti-rabbit IRDye680RD IgG Li-Cor Cat #926-68073; RRID: AB_10954442
Anti-goat IRDye800CW IgG Li-Cor Cat #926-32214; RRID: AB_621846

Validation

All commercially-available antibodies used for flow cytometry were validated by manufacturers. Most antibodies specific to human targets were validated or tested for cross-reactivity with rhesus macaques by manufacturers. In our studies, antibodies were validated in positive controls.

Validation of primary antibodies generated in-house:
• Rabbit hyperimmune serum to murine pneumonia virus (MPV) was produced and validated in-house. Specificity was confirmed by

western blotting, MPV plaque-reduction neutralization assay, and MPV immunoplaque assay (Brock LG, et al.; J Virol 92, (2018) PMID: 29925656).
• Goat hyperimmune serum to the SARS-CoV-2 spike protein was produced and validated in-house by Western blotting, SARS-CoV-2 neutralization assays (BSL3), and immunoplaque assay using other S-expressing viral vectors and empty-vector controls. The hyperimmune serum detects recombinantly-expressed SARS-CoV-2 S protein at the expected size in Western blots, and efficiently neutralizes live SARS-CoV-2 in virus neutralization assays performed in BSL3 on Vero E6 cells (ND50 to USA/WA1/2020: 11.0 log2). [Liu X, et al.;. Proc Natl Acad Sci U S A 118, (2021); PMID: 34876520).
• Anti-S-RBD human CR3022 antibody was produced in-house. Reactivity with the S antigen of the ancestral SARS-CoV-2 strain was validated by immunoplaque assay [Park HS, et al.; NPJ Vaccines 7, 72 (2022); PMID:35764659].

All secondary antibodies are commercially available and validated by the manufacturers.

Eukaryotic cell lines

Policy information about cell lines and Sex and Gender in Research

Cell line source(s)	Vero E6 cells (ATCC CRL-1586) were from ATCC. Vero cells (ATCC CCL-81) were originally from ATCC, amplified under GMP by CRL. The generation of Vero E6 cells stably expressing TMPRSS2 was described previously (Liu X et al., PNAS 2021).
Authentication	Each ATCC cell line was provided with a certificate of analysis. The cell identity was verified by morphology. In addition, the karyotype of Vero cells (ATCC CCL-81) was confirmed by CRL.
Mycoplasma contamination	All cell lines were tested to be negative for mycoplasma.
Commonly misidentified lines (See ICLAC register)	No commonly misidentified cell lines were used.

Animals and other research organisms

Policy information about studies involving animals; ARRIVE guidelines recommended for reporting animal research, and Sex and Gender in Research

Laboratory animals	Rhesus macaques, 12 juvenile or young adults; males, 31-59 months of age.
Wild animals	No wild animals were used in the study.
Reporting on sex	The study was not powered to consider sex as a variable.
Field-collected samples	The study did not involve samples collected from the field.
Ethics oversight	Animal studies were approved by the NIAID Animal Care and Use Committee. The animal experiment was carried out following the Animal Welfare Act and the NIH Guide for the Care and Use of Laboratory Animals in an Association for Assessment and Accreditation of Laboratory Animal Care (AAALAC) accredited facility.

Note that full information on the approval of the study protocol must also be provided in the manuscript.

Plants

Seed stocks	NA
Novel plant genotypes	NA
Authentication	NA

Flow Cytometry

Plots

Confirm that:

☒ The axis labels state the marker and fluorochrome used (e.g. CD4-FITC).

☒ The axis scales are clearly visible. Include numbers along axes only for bottom left plot of group (a 'group' is an analysis of identical markers).

☒ All plots are contour plots with outliers or pseudocolor plots.

☒ A numerical value for number of cells or percentage (with statistics) is provided.

Methodology

Sample preparation	See methods section: Blood was collected in EDTA tubes and PBMCs were isolated by density gradient centrifugation over ficoll. Aliquots of PBMCs were stored in 90% FBS and 10% DMSO in liquid nitrogen. BAL were first filtered through 100 µm filters (Corning, cat# 431752), and cells were collected by centrifugation for 5 min at 544 x g at 10°C. BAL fluid was separated from the cell pellet, aliquoted, snap frozen in dry ice and stored at -80°C for further use. BAL cells were used fresh for evaluation of the S-specific CD4+ and CD8+ T-cell response in the lower airways. Briefly, thawed PBMCs that were rested overnight or freshly collected BAL cells were plated at 1x10^7 cells/ml in 200 µl in 96 well plates in X-VIVO 15 media supplemented with 10% FBS, 1000x Brefeldin (Thermo Fisher Cat# 00-4506-51) and 1000x Monensin (Thermo Fisher Cat# 00-4505-51) diluted 1:1,000, CD107a and CD107b (APC, each diluted at 1:50), and stimulated with the indicated peptide pools at 1µg/ml for 6 h at 37°C with 5% CO2. Spike peptide pools consisted of Peptivator SARS-CoV-2 Prot_S1 (Miltenyi Cat# 130-127-048), Peptivator SARS-CoV-2 Prot_S+ (Miltenyi Cat# 130-127-312), and Peptivator SARS-CoV-2 Prot_S (Miltenyi Cat# 130-127-953) covering the whole spike protein. Nucleocapsid peptide pool consisted of Peptivator SARS-CoV-2 Prot_N (Miltenyi Cat# 130-126-699). After stimulation, cells were centrifuged and stained using a panel of antibodies. The antibodies used for extracellular and intracellular staining were: CD69 (FITC, clone FN50, Biolegend), granzyme B (BV421, clone GB11, BD Biosciences), CD8a (eFluor 506, clone RPAT8, Thermo Fisher), IL-2 (BV605, MQ-17H12, Biolegend), IFNg (BV711, clone 4S.B3, Biolegend), IL-17 (BV785, clone BL168, Biolegend), TNFa (BUV395, clone Mab11, BD Biosciences), CD4 (BUV496, clone SK3, BD Biosciences), CD95 (BUV737, clone DX2, BD Biosciences), CD3 (BUV805, clone SP34-2, BD Biosciences), CD107a (AF647, clone H4A3, Biolegend), CD107b (AF647, clone H4B4, Biolegend), viability Dye eFluor780 (Thermo Fisher), CD103 (PE, clone B-Ly7, eBioscience), CD28 (PE/Dazzle 594, clone CD28.2, Biolegend), Ki-67 (PE-Cy7, clone B56, BD Biosciences), Foxp3 (AF700, clone PCH101, Thermo Fisher).
Instrument	BD FACSymphony A5 (BD Biosciences)
Software	Flowjo software version 10
Cell population abundance	At least 85,000 events were acquired for each sample.
Gating strategy	B cells: Live cells were gated using the live/dead staining and the B-cell marker CD20. Lymphocytes were further identified based on forward and side scatter areas. Next, singlets were identified with a first gate based on forward scatter height and forward scatter area followed by a second gate based on side scatter height and side scatter area. An additional live/dead gate was used to exclude any remaining dead cells as well as CD3+, CD14+, and CD16+ cells. Naïve (IgD+) B cells were also removed from the analysis. S-specific B cells were identified within the live, single, CD3-, CD14- CD16- CD19+ CD20+ IgD- CD95+/- B-cell population using tagged RBD and S-2P protein probes. RBD and/or S-2P-binding B cells were further evaluated for additional phenotypic analysis.<br>T cells: Live cells were first gated based on a live/dead staining and forward scatter area. Next, lymphocytes were identified based on forward and side scatter areas. Then, singlets were selected and an additional live/dead gating was performed to exclude any remaining dead cells. The live single CD3+ IFNgamma+ T-cells were next gated using CD3 and IFNgamma. As CD3 expression can be down-regulated on activated T-cells, a wide CD3 gate was applied. IFNgamma+ CD4+ or IFNgamma+ CD8+ T-cells were next identified using a CD4 or CD8 antibody. An additional CD8 or CD4 gating was performed to remove potentially remaining CD8+ or CD4+ T-cells from the CD4+ or CD8+ T-cell populations, respectively. Finally, non-naïve, non-regulatory CD4+ or CD8+ T-cells were identified using CD95 and Foxp3, respectively. The phenotypic analyses described in Fig. 5, 6, S4, and S6 were performed on live single CD3+ CD4+ CD8- CD95+ Foxp3- or live single CD3+ CD8+ CD4- CD95+ Foxp3- T-cells.

☒ Tick this box to confirm that a figure exemplifying the gating strategy is provided in the Supplementary Information.
